# A systematic investigation reveals that Ishihara et al.’s (2008) STEARC effect only emerges when time is directly assessed

**DOI:** 10.1038/s41598-022-23411-6

**Published:** 2022-11-05

**Authors:** Alberto Mariconda, Valter Prpic, Serena Mingolo, Fabrizio Sors, Tiziano Agostini, Mauro Murgia

**Affiliations:** 1 Department of Life Sciences, University of Trieste, Trieste, Italy; 2 Department of Philosophy and Communication Studies, University of Bologna, Bologna, Italy; 3 Institute for Psychological Sciences, De Montfort University, Leicester, UK

**Keywords:** Psychology, Human behaviour

## Abstract

The Spatial–TEmporal Association of Response Codes (STEARC) effect (Ishihara et al. in Cortex 44:454–461, 2008) is evidence that time is spatially coded along the horizontal axis. It consists in faster left-hand responses to early onset timing and faster right-hand responses to late onset timing. This effect has only been established using tasks that directly required to assess onset timing, while no studies investigated whether this association occurs automatically in the auditory modality. The current study investigated the occurrence of the STEARC effect by using a procedure similar to Ishihara and colleagues. Experiment 1 was a conceptual replication of the original study, in which participants directly discriminated the onset timing (early vs. late) of a target sound after listening to a sequence of auditory clicks. This experiment successfully replicated the STEARC effect and revealed that the onset timing is mapped categorically. In Experiments 2, 3a and 3b participants were asked to discriminate the timbre of the stimuli instead of directly assessing the onset timing. In these experiments, no STEARC effect was observed. This suggests that the auditory STEARC effect is only elicited when time is explicitly processed, thus questioning the automaticity of this phenomenon.

In the last 30 years, the scientific literature has well documented how people cognitively associate space and numbers along a left-to-right oriented mental number line [MNL^2^], with small numbers located on the left and large numbers on the right. The existence of this spatial-numerical association was first demonstrated by Dehaene, Bossini and Giraux^3^, with the so called Spatial-Numerical Association of Response Codes (SNARC) effect. The SNARC effect is characterized by faster left-key responses to small numbers (e.g., 1) and faster right-key responses to large numbers (e.g., 9), within a given numerical interval (e.g., 1–9). This effect has been reported in a variety of tasks, configurations, and sensory modalities^4^. Even tough, it has been widely replicated, the nature of the SNARC effect is still largely debated^5,6^.

Similar to numbers, non-numerical quantities can be spatially coded, eliciting other “SNARC-like” effects. In the visual modality, SNARC-like effects have been observed for luminance^7^, size of pictorial figures^8,9^, angle magnitude^10^, music notation^11–13^ and facial expressions of emotions^14–16^. In the auditory modality, SNARC-like effects have been reported for pitch^17–21^ and loudness^22,23^. Moreover, recent studies showed similar effects also for somatosensory information, such as weight^24,25^ and vibrotactile stimuli^26^.

Accumulating evidence suggests that temporal events can be spatially represented as well^1,27,28^. In fact, similar to numbers, time flow can be mapped on a mental timeline (MTL^1^) oriented from left to right. Consequently, the congruency between temporal information along this MTL and manual response side should facilitate response execution, eliciting a SNARC-like effect. However, the left-to-right orientation should not be seen as universal, indeed it is well-known that SNARC and SNARC-like effects can be modulated by various factors such as the writing/reading system^29^ or manipulations in working memory^30^. Notably, studies on spatial–temporal associations can be classified in two categories: studies that focused on the semantic processing of time, and studies that focused on the perceptual processing of time. In the first category, verbal stimuli that refer to the past or the future (e.g., "yesterday," "tomorrow", "next week”) are typically used. These kinds of studies found a SNARC-like effect, with faster left-hand responses to past-related words and faster right-hand responses to future-related words^31,32^. However, the present work will focus on the second category, namely on perceptual stimuli.

The second category of studies uses non-verbal stimuli whose temporal duration can be manipulated. For instance, Vallesi et al. [Experiment 1^33^] found a SNARC-like effect when participants judged the temporal duration of a visual stimulus presented on a screen, namely a fixation cross. Participants showed faster left-hand responses when the fixation cross lasted 1 s, and faster right-hand responses when it lasted 3 s. In another study by Topić, Stojić and Domijan^34^, the authors presented a visual target alone for short/long durations and then asked participant to detect the same target in a search array, using a go/no-go procedure. They found that the visual search strategies of participants were spatially influenced by the duration of the target preceding the search array, revealing spatial–temporal associations along the vertical and diagonal axes. Similar associations between space and temporal stimuli have been reported in the auditory modality as well, using different paradigms^27,28^.

One of the most influential studies on spatial–temporal associations in the auditory modality is the work by Ishihara et al.^1^, which revealed the Spatial–TEmporal Association of Response Codes effect (STEARC effect). The authors asked participants to report whether the onset timing of a target stimulus (auditory click) was earlier or later than a sequence of reference sounds (7 auditory clicks) which were equally spaced in time. The inter-onset interval (IOI) of the reference sequence was 500 ms, and the onset timing of the target was experimentally manipulated to be “early” or “late” by 43% of the IOI; thus, the targets could appear 215 ms earlier or later than the expected beat. Participants showed faster left-hand responses when the target was early, and faster right-hand responses when the target was late. It is noteworthy that the task employed in this study required participants to explicitly assess the timing of the target.

The task typically employed in SNARC/SNARC-like studies can be classified in two categories: direct and indirect tasks^35^. In direct tasks, participants are asked to explicitly judge a property of the target stimuli that is relevant for the study (e.g., number magnitude); usually, the judgment requires the comparison between a target and a reference stimulus. Conversely, in indirect tasks, participants are asked to judge a property of the target that is not relevant for the study (e.g., number parity) while the relevant one (e.g., number magnitude) is expected to be implicitly processed. As for spatial–temporal associations, there is evidence for the occurrence of these effects in direct tasks with both verbal^36^ and perceptual stimuli^1^. Conversely, results are less clear for indirect tasks: negative results have been reported for verbal stimuli^34^, while the literature is scarce for perceptual stimuli. To the best of our knowledge, the only studies employing indirect tasks with perceptual (visual) stimuli were the one by Topić et al.^34^, which showed the occurrence of spatial–temporal associations, and the one by Dalmaso et al.^37^, which showed no STEARC effect. However, there are no studies on spatial–temporal associations employing indirect tasks in the auditory modality. Furthermore, Topić et al.^34^ adopted a methodological approach that largely differs from classical SNARC-like paradigms. Thus, further research is needed to determine whether the STEARC effect can be elicited by using indirect tasks.

In the present study we investigated the STEARC effect by using auditory perceptual stimuli, employing both direct and indirect tasks. In particular, we firstly used a procedure similar to Ishihara et al.^1^, aiming to conceptually replicate the study and test the robustness of the STEARC effect employing a direct task, as in the original study. It is noteworthy that, although its popularity, this study has never been replicated. Moreover, we conducted a set of experiments with modified versions of the paradigm employed by Ishihara et al., with the aim to test whether the STEARC effect still occurs in indirect tasks.

## Experiment 1

In Experiment 1, the occurrence of the STEARC effect along the MTL was tested using a direct task. A procedure similar to the one employed by Ishihara et al.^1^ was used, namely asking participants to judge whether the onset timing of the target stimuli occurs earlier or later than the expected beat. However, different from the original study, which employed only two onset timing conditions, in the present experiment four onset timing conditions were used.

According to the findings by Ishihara et al.^1^, we expected faster left-hand responses for early targets and faster right-hand responses for late targets. Furthermore, similar to Vallesi et al. [Experiment 5^33^] we expected to observe a distance effect, with faster responses for the “extreme targets” (i.e., the fastest and the slowest onset timing), and slower responses for the two “central targets”. Finally, we were interested in exploring whether stronger spatial–temporal associations occur for the extreme targets compared to the central targets, which would reveal a mapping along a continuum, or whether extreme and central targets show similar levels of spatial associations (categorical mapping).

## Methods

### Participants

Thirty-five italian university students (M = 13, F = 22; age: M = 25.77, SD = 3.80) from the University of Trieste, with left-to-right reading/writing direction, were recruited for this experiment. All of them received academic credits for their participation to the experiment. Before starting the experiment, all participants completed a questionnaire in which they reported their handedness, any vision or hearing impairment, and whether their psychophysiological state was affected by alcohol consumption or insufficient sleep in the last 24 h. One participant was left-handed; all of them reported to have normal hearing, normal or corrected to normal vision and that their psychophysiological state was not affected by alcohol consumption or insufficient sleep in the last 24 h^38^. All participants of this study signed a written informed consent before the beginning of the experiments. The study was conducted in accordance with the ethical standards established by the Declaration of Helsinki and was approved by the University of Trieste Ethics Committee.

### Apparatus and stimuli

The experiment was designed and run through the Psychopy software version 3.0^39^. The experiment was run with a HP laptop with Intel Core i7 (RAM: 16 Gb). A five-button serial response box was employed to collect participants’ responses.

Auditory click sounds were used as stimuli (each click sound consisted of a 1000 Hz tone with 200 ms duration). The inter-onset interval (IOI) was 500 ms (for the first seven clicks). The timing of the eighth click that served as target was experimentally manipulated by decreasing or increasing the duration of the last IOI by 66.67% (− 333 ms; + 333 ms) or 33.33% (− 167 ms; + 167 ms). Therefore, the early targets were presented 167 ms (“early 1”) or 333 ms (“early 2”) after the end of the reference sequence, while the late targets were presented 667 ms (“late 1”) or 833 ms (“late 2”) after the end of the reference sequence. Participants listened to the stimuli at a comfortable level through the headphones (Sony MDR-XB950/B) connected to the computer.

### Procedure

Participants were tested individually in a quiet laboratory room. They were asked to sit in front of the response box and the screen, at a comfortable distance to read the instructions. The participants’ body was aligned to the midline of the response box. The experiment had a total duration of about 50 min and was carried out with eyes closed. It consisted of 4 alternate blocks (A-B-A-B or B-A-B-A) counterbalanced among participants. In each block there were a practice session and an experimental session (the task was the same in both sessions). Before starting each experimental session, participants performed the corresponding trial session to become familiar with the task, where feedback was provided on the correctness of the answer (“Correct!” or “Wrong!”).

Each participant heard a sequence of eight auditory clicks in succession. Participants were instructed to listen to the entire click sequences in each session and to indicate whether the onset timing of the target (8th click) was earlier or later than the expected beat, by pressing one of the two response buttons as quickly and accurately as possible. During each block, the left index was placed on the left button while the right index was placed on the right button. In detail, in block A, participants were instructed to press the left key to respond to early targets, and the right key to late targets; in block B, this stimulus–response (S–R) mapping was reversed. An additional “no-go” condition (without the 8th click) was added to induce participants to wait for the late target as in the Ishihara et al.’^1^ original study. In the no-go condition, participants were instructed to press no key; a warning sound was presented if participants pressed a key in these trials. After the participant's response, the inter-trial interval was 2 s for all trials.

In total, there were four alternate blocks (A-B-A-B or B-A-B-A), each consisting of 50 randomized trials (40 targets and 10 no-go) for each S–R condition. Each participant completed a total of 200 experimental trials with 80 trials for early targets, 80 trials for late targets and 40 trials for the no-go condition.

### Data analysis

The no-go condition and the incorrect responses (5.12%) were excluded from the analyses. In addition, outliers (2.05%) were also removed according to the criteria adopted by Ishihara et al.^1^: response times shorter than 120 ms or longer than the average response times of each participant plus 3 standard deviations were excluded.

First, a 2 × 4 Repeated Measures (ANOVA) was performed on response times. The independent variables were two: Response hand (left vs. right) and Onset timing of the target (early 1 vs. early 2 vs. late 1 vs. late 2). Second, the difference between right-hand and left-hand response times (RT) was computed [dRT = RT (right hand) − RT (left hand)] for the four conditions of the targets (onset timing of the target: early 1; early 2; late 1; late 2), in order to examine the individual slopes for each participant. Faster left-hand responses resulted in positive dRTs, whereas faster right-hand responses resulted in negative dRTs. Then, a linear regression analysis was performed, with dRTs as dependent variable, to obtain B coefficients for each participant; negative values for B coefficients indicate a negative slope (SNARC). Finally, a one-sample *t-*test was performed to test whether the mean of coefficients B significantly deviated from zero.

In addition, a paired-sample *t-*test was computed, in order to analyze the distance effect, comparing the absolute response times of the two “extreme targets” (i.e., the fastest and the slowest, thus early 1 and late 2, respectively) with the two “central targets” (i.e., early 2 and late 1). A similar comparison was conducted on the strength of spatial–temporal associations for the extreme vs. the central targets, to investigate whether the mapping was categorical or along a continuum. In this case, the extreme values were the average of the dRTs for early 1 and late 2 (the latter with the reversed sign), while the central values were the average of the dRTs for early 2 and late 1 (the latter with the reversed sign).

### Results and discussion

**Figure 1:**
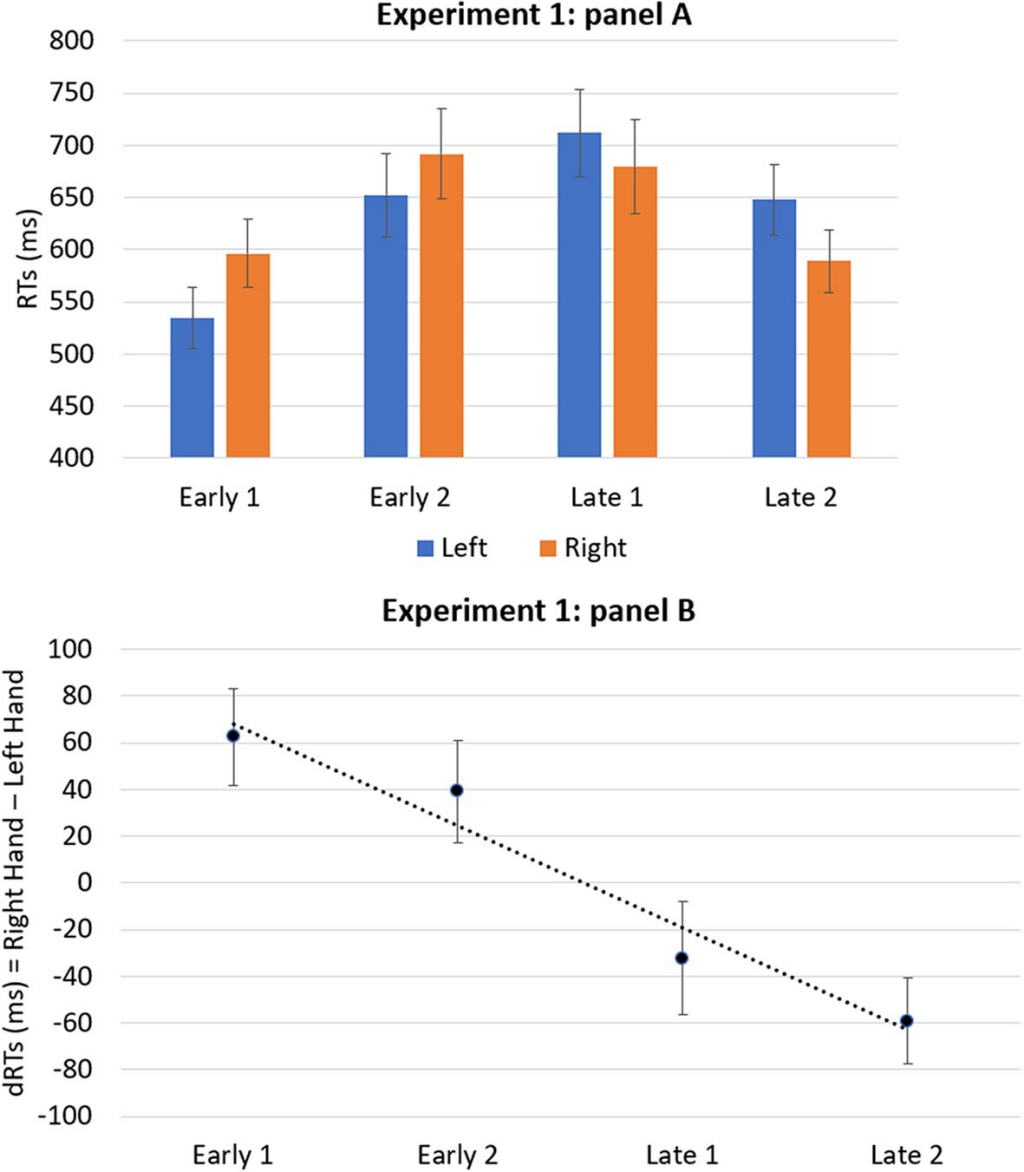
The panel (**A**) shows the mean of response times of the left and right hands, for the four onset timing of the target (early 1, early 2, late 1, late 2). The panel (**B**) shows the mean dRTs (right key–left key) for the four onset timing of the target. Positive differences indicate faster left-key responses; negative differences indicate faster right-key responses. Errors bars indicate standard error of the mean.

The ANOVA revealed a significant main effect for the onset timing [*F*(3, 102) = 28.80; *p* < 0.001; *η*_*p*_^2^ = 0.459], while the main effect for hand was not significant [*F*(1, 34) = 0.199; *p* = 0.658; *η*_*p*_^2^ = 0.006]. A significant interaction between hand and onset timing emerged [*F*(3, 102) = 6.98; *p* < 0.001; *η*_*p*_^2^ = 0.170]. The analysis of the one-sample *t-*test revealed that the mean of coefficients B significantly deviated from zero [*t*(34) = − 3.27; *p* < 0.005;* d* = − 0.552]. Results are reported in Fig. 1. A paired sample *t*-test revealed a distance effect, indeed the absolute response times for the extreme onset timing (M = 592 ms; SD = 174) were significantly faster compared to those for the central onset timing (M = 680 ms; SD = 238) [*t*(34) = − 7.375; *p* < 0.001; *d* = − 1.25]. Moreover, as for the strength of spatial–temporal associations (calculated on dRTs), the difference between extreme (M = 60 ms; SD = 103) and central (M = 38 ms; SD = 104) onset timing did not reach statistical significance [*t*(34) = 1.68; *p* = 0.103; *d* = 0.283].

The results of Experiment 1 were consistent with the study by Ishihara et al.^1^ and showed a SNARC-like effect with faster left-hand responses to early stimuli and faster right-hand responses to late stimuli, when a direct task is employed. This effect was confirmed by both the ANOVA and the one-sample *t*-test on B regression coefficients. Moreover, a paired samples *t*-test revealed a significant distance effect, with extreme onset timing being responded faster than central ones, probably because they are easier to discriminate. Finally, although descriptive statistics apparently indicate stronger spatial–temporal associations for the extreme vs. central onset timing, the lack of statistical significance seem to support a more categorical rather than continuous mapping of time.

In order to investigate whether similar spatial–temporal associations still occur when time is not a relevant dimension for the task (i.e., with indirect tasks), we conducted the Experiment 2.

## Experiment 2

In Experiment 2 we used an indirect task to investigate whether an automatic activation of the spatial–temporal association occurs. To do so, we used a procedure similar to that used in Experiment 1, but we asked participants to identify the timbre of auditory targets (metronome or electronic sound) instead of judging whether they occurred earlier or later than expected (the targets could be early or late as in Experiment 1, but the onset timing was irrelevant to the task). This task was chosen because timbre is a property of the stimuli easy to discriminate and was previously employed in SNARC-like literature, revealing spatial associations for pitch and loudness^18,21,40^.

## Method

### Participants

Thirty-five Italian university students (M = 5; F = 30; age: M = 21.77, SD = 3.30) were recruited for this experiment (none of them had been recruited for the previous experiment); five of them were left-handed. The sample has the same characteristics as the one of Experiment 1 (reported normal hearing, left-to-right reading/writing direction, reported no alcohol consumption or insufficient sleep, received academic credits).

### Apparatus and stimuli

The apparatus used in Experiment 2 was the same as the one used in the Experiment 1. As in the previous experiment, auditory click sounds were used as stimuli. In detail, 7 sequential click sounds were used as neutral stimuli (each click sound had a frequency of 1000 Hz and lasted 200 ms), while the targets were either a classic wooden metronome (1123 Hz) or an electronic sound (386 Hz) of the same duration.

### Procedure

The procedure was similar to the one used in the Experiment 1. The only differences were that the first 7 auditory clicks served as neutral sequence and the task required participants to judge whether the target (i.e., 8th sound) was a metronome or an electronic sound. The timbre judgement task was similar to that previously employed in SNARC-like literature^21^. As in the previous experiment, the targets could appear early or late, but the onset timing was irrelevant to the task. In the block A, the left response was assigned when the target was the beat of metronome and the right response was assigned when the target was the electronic sound, irrespective of their early or late occurrence. In the block B, this mapping was reversed.

Each participant completed a total of 200 experimental trials: 40 repetitions for the early metronome (20 for early 1 and 20 for early 2), 40 repetitions for the late metronome (20 for late 1 and 20 for late 2), 40 repetitions for the early electronic sound (20 for early 1 and 20 for early 2), 40 repetitions for the late electronic sound (20 for late 1 and 20 for late 2) and 40 repetitions for the no-go.

### Data analysis

Data analyses were the same as in Experiment 1, with the only difference that the ANOVA was 2 × 2 × 4, given that the timbre was added as factor. Incorrect responses (3.11%) and outliers (1.77%) were excluded from the analyses.

### Results and discussion

**Figure 2:**
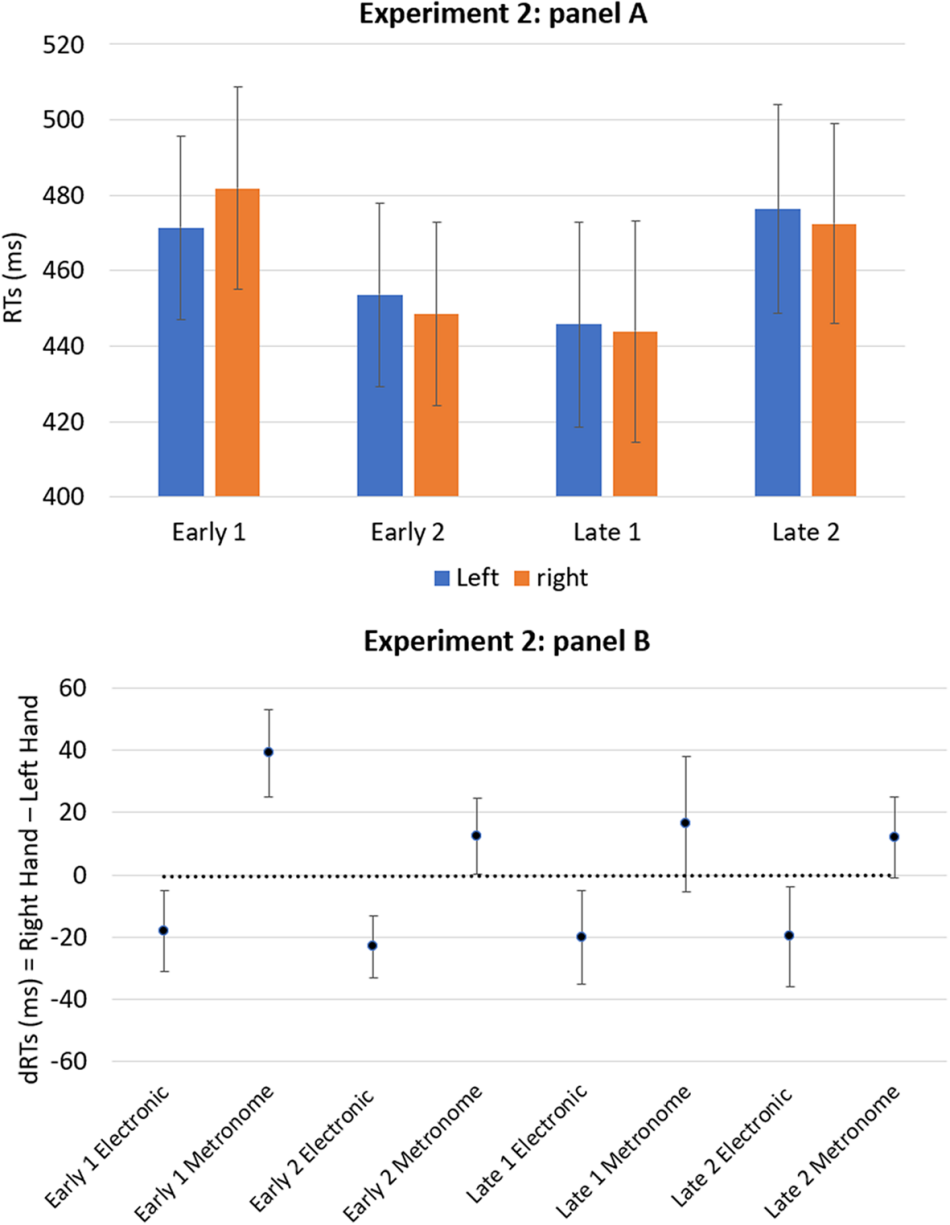
The panel (**A**) shows the mean of response times of the left and right hands for the four onset timing of the target (early 1, early 2, late 1, late 2). The panel (**B**) shows the mean dRTs (right key–left key) for each condition of the target. Positive differences indicate faster left-key responses; negative differences indicate faster right-key responses. Errors bars indicate standard error of the mean.

The ANOVA revealed a significant main effect for the Onset timing [*F*(3, 102) = 8.73; *p* < 0.001; *η*_*p*_^2^ = 0.204] and for the Timbre [*F*(1, 34) = 9.37; *p* < 0.005; *η*_*p*_^2^ = 0.216]. No other main effect or interactions were significant, apart from a marginally significant interaction between Hand and Timbre [*F*(1, 34) = 2.85; *p* = 0.099; *η*_*p*_^2^ = 0.078]. Results are reported in Fig. 2. The analysis of the one-sample *t-*test revealed that the mean of coefficients B did not deviate significantly from zero [*t*(34) = − 0.344; *p* = 0.733;* d* = *− *0.058].

The results of Experiment 2 did not show a SNARC-like effect with faster left-hand responses to early stimuli and faster right-hand responses, independently from the target presented; both ANOVA and one-sample *t*-test indicate that the spatial–temporal association did not occur when temporal information was irrelevant to the task and participants were expected to implicitly process the temporal durations of the stimuli.

For exploratory purposes, separate analyses were also conducted for the two timbres (metronome and electronic sounds), given that a significant main effect for the timbre was found.

For the beat of metronome, the ANOVA only revealed a significant main effect for the Onset timing [*F*(3, 102) = 5.71; *p* < 0.005; *η*_*p*_^2^ = 0.144]. Moreover, the analysis of the one-sample *t-*test revealed that the mean of coefficients B deviated significantly from zero [*t*(34) = − 2.107; *p* < 0.005; *d* = − 0.356]; in this case, the negative mean of the B coefficients that deviated significantly from zero indicates a SNARC-like effect.

For the electronic sound, the ANOVA only revealed a significant main effect for the Onset timing [*F*(3, 102) = 6.56; *p* < 0.001; *η*_*p*_^2^ = 0.162]; Moreover, the analysis of the one-sample *t-*test revealed that the mean of coefficients B did not deviate significantly from zero [*t*(34) = − 0.270; *p* = 0.789; *d* = − 0.045].

Overall, in this experiment we did not find a SNARC-like effect with perceptual stimuli in indirect task, this is consistent with other studies that used verbal stimuli^36^. However, two interesting findings emerged from this experiment. First, the timbre of the targets influenced responses times. Indeed, metronome sounds were responded faster than electronic sounds; this result was not due to the difference in stimulus intensity (dB); in fact, the electronic sound and the metronome had a maximum peak of 79.3 dB and 78.1 dB, respectively. Furthermore, as revealed by the pattern in Fig. 2 (panel B) and the marginally significant interaction between Hand and Timbre, it seems that participants associated electronic sounds with right-key responses and metronome sounds with left-key responses. Second, the negative mean of coefficients B deviated significantly from zero only for the beats of the metronome, suggesting that participants showed evidence of a STEARC effect only with this specific sound. Due to these results, we hypothesized that the processing of the timbre could prevail on the processing of onset timing, thus masking a STEARC effect.

Another explanation for the absence of the STEARC effect is that the perceptual unity of the stimuli could be lost, due to the violation of the Gestalt similarity principle. The similarity principle claims that elements tend to be integrated into groups (perceptual units) if they are similar to each other (as in Ishihara’s original experiment and in our Experiment 1, in which the same sound was always used for both the reference and target stimuli). In Experiment 2 we used different sounds, therefore it is possible that the reference and target stimuli were perceived as different perceptual units; as a result, the temporal information that linked the targets to the initial sound sequence could be lost. A possible solution to strengthen the perceptual unity of stimuli is to use the proximity principle, for which elements tend to be perceived as aggregated into perceptual units if they are near each other^41^. Thus, to strengthen the perceptual unity of stimuli, we decided to speed up the tempo of the sequence by reducing the duration of the IOI that separated both the clicks in the neutral sequence and the onset timing of the target.

Based on these considerations, we designed Experiment 3a speeding up the stimuli according to the proximity principle of Gestalt theory, and Experiment 3b modifying the timbre of the targets.

## Experiment 3a

To strengthen the perceptual unity of the neutral sequence and the target stimulus we decided to reduce the duration of IOI, thus producing a faster beat sequence. We hypothesized that, by reinforcing the perceptual unity of stimuli with the proximity principle, we could enhance the possibility to elicit the STEARC effect.

## Method

### Participants

Thirty-five Italian university students (M = 9; F = 26; age: M = 19.40, SD = 0.81) were recruited for this experiment (none of them had been recruited for the previous experiments); two of them were left-handed. The sample has the same characteristics as those of previous experiments.

### Apparatus and stimuli

The apparatus and stimuli used in Experiment 3a were the same as the one used in the Experiment 2, with only two differences. The first was that inter-onset interval (IOI) for the first 7 auditory clicks was 300 ms (instead of 500 ms). The second was that onset timing of the targets was experimentally manipulated in such a way as to be 1/2 of the IOI (150 ms), 2/3 of the IOI (200 ms), 3/2 of the IOI (450 ms) and 2/1 of the IOI (600 ms).

### Procedure

The procedure was the same as the one used in the Experiment 2.

### Data analysis

Data analyses were the same as in Experiment 2. Incorrect responses (2.66%) and outliers (1.74%) were excluded from the analyses.

### Results and discussion

**Figure 3:**
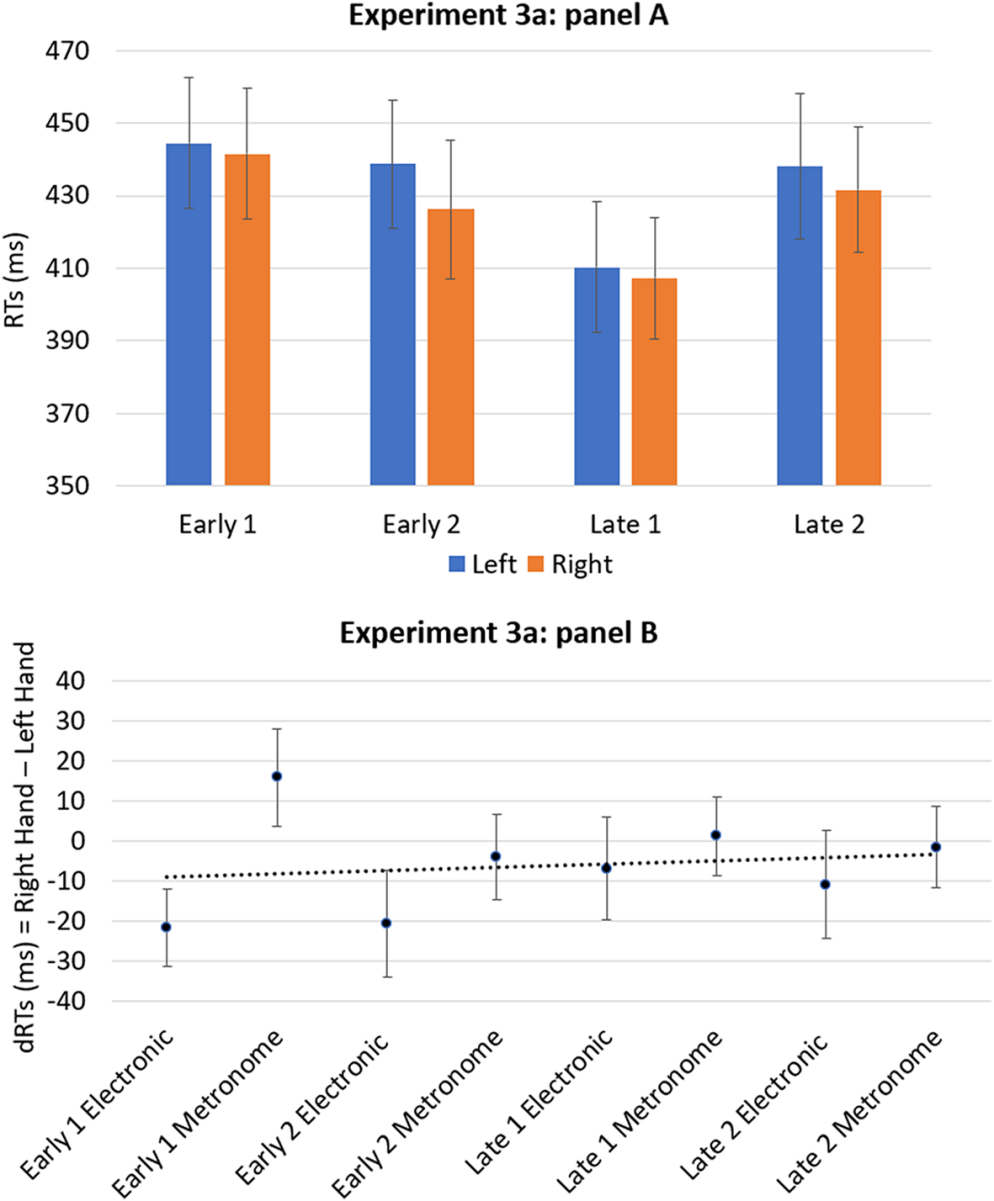
The panel (**A**) shows the mean of response times of the left and right hands for the four onset timing of the target (early 1, early 2, late 1, late 2). The panel (**B**) shows the mean dRTs (right key–left key) for each condition of the target. Positive differences indicate faster left-key responses; negative differences indicate faster right-key responses. Errors bars indicate standard error of the mean.

The ANOVA revealed a significant main effect for the Onset timing [*F*(3, 102) = 13.61; *p* < 0.001 *η*_*p*_^2^ = 0.286] and for the Hands [*F*(1, 34) = 4.87; *p* < 0.005; *η*_*p*_^2^ = 0.125] but did not reveal any other main effect or interaction. The analysis of the one-sample *t-*test revealed that the mean of coefficients B did not deviate significantly from zero [*t*(34) = 0.392;* p* = 0.697;* d* = 0.066]. Results are reported in Fig. 3.

Separate analysis of two target types only revealed significant main effects of the Onset timing for both the metronome [*F*(3, 102) = 8.45; *p* < 0.001; *η*_*p*_^2^ = 0.199] and the electronic sound [*F*(3, 102) = 6.51; *p* < 0.001; *η*_*p*_^2^ = 0.161], but no significant interactions.

Our results indicate the absence of a STEARC effect even when the duration of IOI has been reduced to reinforce the perceptual unity of the temporal sequence, as suggested by the proximity principle. Therefore, our interpretation of this results is that the absence of STEARC was not due to the loss of perceptual unity.

## Experiment 3b

As shown by the results of Experiment 2, the timbre could have masked the STEARC effect. In particular, our data indicates that the target stimuli determined lateralized responses, probably due to their auditory features (independent from the onset timing, participants showed faster left-hand responses with the metronome and faster right-hand responses with the electronic sound). This could be because we did not control for the pitch of target sounds, therefore if a sound was perceived as higher than the other, this could lead to a SNARC-like effect for pitch (left-key responses are faster for low pitch, right-key responses are faster for high pitch)^18,21^. To control for this possible confound we decided to use two target sounds that clearly differ in timbre (piano and violin) but have the same pitch height.

## Method

### Participants

Thirty-five Italian university students (M = 8; F = 27; age: M = 20.94, SD = 2.55) were recruited for this experiment (none of them had been recruited for the previous experiments); two of them were left-handed. The sample has the same characteristics as those of previous experiments.

### Apparatus and stimuli

The apparatus used in Experiment 3b was the same as the one used in previous Experiment 2. As in the Experiment 2, we used 7 click sound (1000 Hz tone, lasting 200 ms each) as neutral stimuli, while for the targets we used the sounds of a piano or a violin with the same pitch (G5#, 386 Hz), instead the metronome or the electronic sounds. The timbre sounds used (piano vs. violin discrimination) were the same as in a previous study by Chang and Cho^40^.

### Procedure

The procedure was the same as the one used in Experiment 2 and 3.

### Data analysis

Data analyses were the same as in Experiment 2 and 3. Incorrect responses (3.71%) and outliers (2.65%) were excluded from the analyses.

### Results and discussion

**Figure 4:**
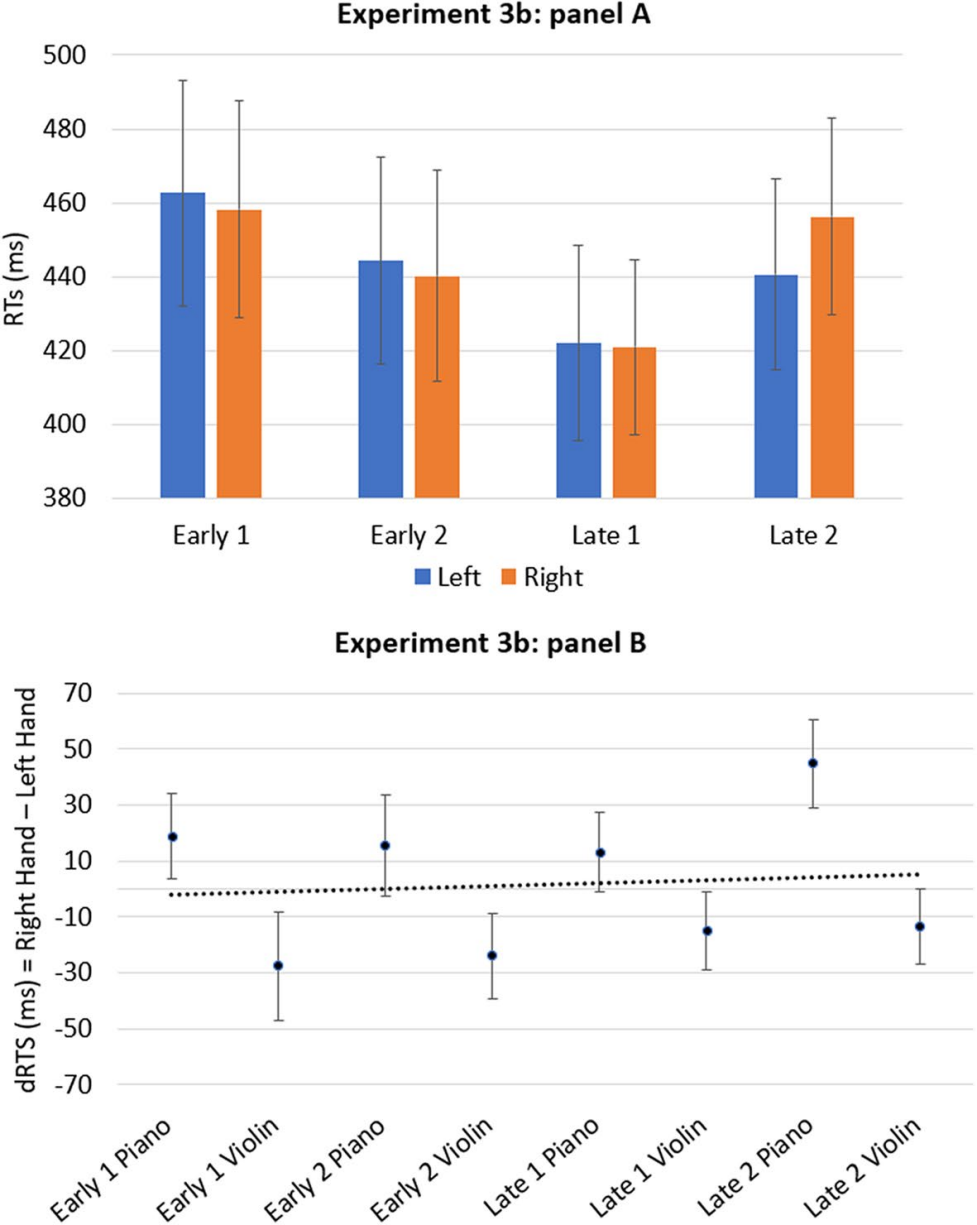
The panel (**A**) shows the mean of response times of the left and right hands for the four onset timing of the target (early 1, early 2, late 1, late 2). The panel (**B**) shows the mean dRTs (right key–left key) for each condition of the target. Positive differences indicate faster left-key responses; negative differences indicate faster right-key responses. Errors bars indicate standard error of the mean.

The ANOVA revealed only a significant main effect for the Onset timing [*F*(3, 102) = 10.49; *p* < 0.001; *η*_*p*_^2^ = 0.236]. No other significant main effects or interactions were found. However, a marginally significant interaction between Hand and Timbre was found [*F*(1, 34) = 3.62; *p* = 0.066; *η*_*p*_^2^ = 0.096]. The analysis of the one-sample *t-*test revealed that the mean of coefficients B did not deviate significantly from zero [*t*(34) = 0.627; *p* = 0.535;* d* = 0.106 ]. Results are reported in Fig. 4.

Separate analysis of two target types revealed only significant main effect of the Onset timing for both Piano [*F*(3, 102) = 4.87; *p* = 0.003; *η*_*p*_^2^ = 0.125] and Violin [*F*(3, 102) = 5.75; *p* = 0.001; *η*_*p*_^2^ = 0.145]. In addition, for the piano, the analysis of the one-sample *t-*test revealed that the mean of coefficients B deviated significantly from zero [*t*(34) = 2.04; *p* < 0.05; *d* = 0.345].

The results of Experiment 3b replicated the one of Experiment 2 by revealing a marginally significant interaction between Hand and Timbre. This evidence suggests that participants associate timbre with space, although we controlled for pitch height. Again, globally no evidence of a STEARC effect emerged.

When separate analyses were performed for the different timbres, a significant t-test emerged only for the piano. However, the direction of this association was not consistent with the one expected from the STEARC effect (see Fig. 4).

## General discussion

This study investigated the STEARC effect^1^, a classical demonstration of the spatial mapping of time. This effect consists in faster left-key responses for early onset timing and faster right-key responses for late onset timing. Despite its popularity, this effect has not been replicated or further investigated in the auditory modality. In particular, there is no evidence that onset timing can be automatically associated with space, due to the scarce literature employing indirect tasks. Our results show a STEARC effect only with a direct task, while no clear evidence of an association for onset timing was revealed in indirect tasks.

Experiment 1 was a conceptual replication of the study by Ishihara et al.^1^, which consisted in a direct task where participants judged whether the onset timing of the target occurred early or late compared to a sequence of reference sounds. The main difference from the original study was that we manipulated onset timing by using four stimuli (two early and two late) instead of two (one early and one late). This manipulation allowed us to assess the presence of a distance effect, with faster responses for the “extreme targets” (i.e., the fastest and the slowest onset timing), and slower responses for the two “central targets”. However, the presence of the distance effect does not clarify the nature of spatial–temporal associations (i.e., categorical vs. continuous), given that in this case the distance effect could derive from the different ease of target discrimination. Conversely, no significant difference was found for the strength of spatial–temporal associations for the extreme vs. central targets, supporting a more categorical rather than continuous mapping of time, consistently with another study by Ishihara et al.^42^, as well as other SNARC studies employing direct tasks^4^.

Experiment 2 was designed to investigate whether the activation of a spatial–temporal association can occur automatically in a task where time is not a relevant dimension. We adapted the procedure used in Experiment 1 by employing an indirect task, namely a timbre judgment. In this task participants were asked to judge the timbre of the target sound (metronome or electronic) while, different from Experiment 1, they were not required to directly process onset timing. No evidence of a STEARC effect emerged with this task. However, when separate analyses were performed for the two timbre sounds, a significant one-sample *t*-test on B regression coefficients suggested a possible SNARC-like pattern, although this was not supported by the ANOVA. Another interesting finding that emerged was a marginally significant interaction between hand and timbre, which indicating that electronic sounds were responded faster with the right key and metronome sounds were responded faster with a left key.

Globally, results from Experiment 2 suggest that the STEARC effect is not revealed when an indirect task is employed. This was the first attempt to investigate a spatial–temporal association with auditory perceptual stimuli. Our finding is consistent with other studies that used verbal stimuli^36^, while only one study^34^ showed evidence of a spatial–temporal association when an indirect task was employed. These results indicate that the spatial–temporal association is not automatic and that it is only elicited when time is directly processed. However, two alternative explanations could not be excluded. One is that by modifying the timbre sound of the stimuli the perceptual unity between the reference sequence and the target was lost. In fact, this could be due to a violation of the Gestalt similarity principle (different sounds could be perceived as not being part of the same unity). In accordance with the proximity principle, to reinforce the perceptual unity of the temporal sequence, we sped up the tempo by reducing the duration of IOI. A second alternative explanation was that an association between the pitch of different sounds and space could mask the STEARC effect. To control for the possible confounding variable of pitch, we used tonal stimuli with the same pitch height (G#5) and different timbre (violin and piano). Based on these alternative explanations, we designed Experiments 3a and 3b.

Results from Experiment 3a replicated the ones from Experiment 2, thus showing that a STEARC effect is not elicited when onset timing is not directly assessed. In this experiment we reinforced the perceptual unity of the stimuli through the proximity principle, for which elements tend to be perceived as aggregated into perceptual units if they are near each other. Therefore, our first alternative explanation that the absence of the STEARC effect could be due to a loss of perceptual unity was not supported. Although we are confident that we reinforced the perceptual unity of the stimuli by increasing their speed, we cannot exclude that the violation of the similarity principle still prevented the perception of the sequence as a unity; we acknowledge this is a limitation of the present experiment.

Similarly, the results from Experiment 3b also showed no evidence of a STEARC effect, although pitch height was controlled. In addition, when separate analyses were performed for the two timbres, a significant one-sample *t*-test on B regression coefficients emerged for the piano sounds. However, the direction of this pattern was opposite to the one expected from the STEARC effect and, more importantly, this was not supported by the ANOVA. Similar to Experiment 2, a marginally significant interaction between hand and timbre emerged. Since pitch height was kept constant in Experiment 3b, we can exclude that this effect is due to an association between pitch and response location^18,21^. Thus, this finding seems to indicate a possible association between timbre sound and response location. Specifically, piano was responded faster with the left key and violin was responded faster with the right key. As timbre has neither magnitude nor ordinal properties, this association seems to mimic a MARC-like effect, which is commonly found in parity judgment with numerical stimuli^43–45^. Although this effect was only marginally significant, this response location advantage was consistent throughout both stimuli and experiments (Experiment 2 and Experiment 3b). Based on these considerations, we cannot exclude that the timbre could have masked the STEARC effect, or that timbre judgment—which is not based on time-related properties—might not be effective in eliciting the spatial–temporal association. The limited access of this task to the timing-related system might be the reason why the STEARC effect and, more in general, the SNARC-like effects do not systematically occur in indirect tasks.

Overall, Experiment 3a and 3b seem to exclude two possible alternative explanations for the absence of the STEARC effect in indirect tasks, further suggesting that the spatial–temporal association is not automatic and it is only elicited when time is directly processed. The automaticity of spatial associations has been largely debated in literature, and empirical evidence supports it for numbers. Indeed, many studies^46,47^ show that the SNARC effect is present even when numerical information is irrelevant to the task. Conversely, based on our study, the spatial associations for time seem not to be automatic but task-dependent. This evidence is similar to the findings by Dalmaso et al.^37^ who failed to find a vertical STEARC effect in the visual modality, when time was an irrelevant dimension for the task. Interestingly, our results seem in contrast with those by Topić et al.^34^, but the different paradigms used by the authors could be the reason of this apparent conflict. On a broader perspective, the lack of automaticity of spatial associations for time seems similar to that found for other non-numerical magnitudes, as shown by the majority of previous studies (for a review see the work by Macnamara et al.^48^; although there are interesting exceptions^7,49^).

Future studies should further investigate a novel phenomenon revealed by our study, namely the association between timbre sounds and space. Indeed, several studies that previously used timbre judgment did not reveal such association^18,20,21^. Although in our study we controlled for pitch height, we did not control for brightness which generally covariates with pitch height and could influence the spatial association^19^. Future studies should also investigate the STEARC effect with vertical and sagittal responses. Although Ishihara et al.^1^ failed to show an association between onset timing and the sagittal space, no studies have tested the vertical space by using this paradigm (but see the study by Dalmaso and colleagues for visual stimuli^37^). Furthermore, recent studies found evidence of a compatibility effect between time and the vertical space by using a different paradigm^34^, therefore more studies are needed to further investigate this phenomenon beyond the horizontal axis.

## Conclusion

In previous literature, spatial–temporal associations has only been established with direct tasks (with both verbal and perceptual stimuli), while no studies employed indirect tasks with perceptual (auditory) stimuli. To further investigate this phenomenon, the current study investigated the occurrence of the STEARC effect^1^ in both direct and indirect tasks, across four experiments.

Consistently with the original work by Ishihara et al.^1^, we successfully replicated the STEARC effect by employing a direct task in Experiment 1. Furthermore, although we found a significant distance effect with extreme onset timing being responded faster than central onset timing, the spatial–temporal association seems to be mapped categorically rather than along a continuum.

Conversely, when we employed indirect tasks, the STEARC effect was not elicited, consistently with other studies that used verbal stimuli^36^. In fact, Experiment 2 shows that when temporal information is irrelevant for the task and participants can only implicitly process the temporal durations of auditory stimuli, the spatial–temporal association did not occur. This was further confirmed in Experiment 3a and 3b, where IOI and pitch were manipulated to control for possible confounding effects. Taken together, the results show that the auditory STEARC effect is only elicited when temporal information is explicitly processed, suggesting that it is not due to the automatic processing of time.

## Data Availability

The datasets generated and/or analysed during the current study are available in the “OSF Storage” repository, https://osf.io/y5hsw/?view_only=afad2d40f9e2404ca70d3461d6dbb711.
